# Pre-Existing Cytokine and NLRP3 Inflammasome Activation and Increased Vascular Permeability in Diabetes: A Possible Fatal Link With Worst COVID-19 Infection Outcomes?

**DOI:** 10.3389/fimmu.2020.557235

**Published:** 2020-11-23

**Authors:** Vaia Lambadiari, Foteini Kousathana, Athanasios Raptis, Konstantinos Katogiannis, Alexander Kokkinos, Ignatios Ikonomidis

**Affiliations:** ^1^ 2^nd^Department of Internal Medicine, Research Unit and Diabetes Center, Medical School, Attikon University Hospital, National and Kapodistrian University of Athens, Athens, Greece; ^2^ 2^nd^Cardiology Department, Attikon University Hospital, Medical School, National and Kapodistrian University of Athens, Athens, Greece; ^3^ 1^st^Department of Propaedeutic Internal Medicine, Medical School, Diabetes Center, Laiko Hospital, National and Kapodistrian University of Athens, Athens, Greece

**Keywords:** cytokine, diabetes, glycocalyx, inflammasome, inflammation

## Introduction

SARS-CoV-2, a novel RNA contaminating coronavirus emerged in Wuhan, China at the end of 2019 (COVID-19). In March 11, 2020 the World Health Organization declared it a pandemic disease. Since April 14, 2010 the pandemia has been spread to 3,170,474 people and led to 220,324 deaths (1). Findings from a February report from the Chinese Center for Disease Control and Prevention have shown that of 44,672 cases the overall case-fatality rate (CFR) was 2.3%. CFR was elevated among COVID-19 patients with comorbidities, such as cardiovascular disease (10.5%) and diabetes (7.3%) (2).

Patients with uncontrolled diabetes may be at extra risk of COVID-19 mortality or complications of the disease. The pathophysiology remains unknown, although it is of great interest because of the great prevalence of diabetes; In 2019, 463 million of adults 20–79 years old were living with diabetes, and by 2045 this number is suggested to rise to 700 million (3).

## COVID-19: Possible Mechanisms of Increased Risk Complications in Diabetic Patients

### The Role of NLRP3 Inflammasome in COVID-19 and Its Complications

In COVID-19 the immune system may react with an exacerbation of cytokine production known as a “cytokine storm” which leads to hyperinflammation and secondary hemophagocytic lymphohistiocytosis (sHLH) or else Macrophage Activation Syndrome (MAS). This is characterized by fulminant, fatal hypercytokinemia, multiple organ failure, Acute Respiratory Distress Syndrome (ARDS), and sepsis (4). NLRP3 inflammasome seems to play an important role in these complications (5).

Particularly, numerous studies have implicated the NLRP3 inflammasome and IL-1β in mediating inflammation during lung injury and ARDS (6, 7). In ARDS/ALI, IL-1β is one of the most biologically active proinflammatory cytokines in the lungs, while inflammasome hyperactivation participates in both ARDS and mechanical ventilation acute lung injury. IL-1β levels are shown to be elevated in plasma and bronchoalveolar fluid of patients with ARDS (8). In patients with ARDS infected with MERS-CoV and SARS-CoV, IL-1β, IL-6, and IL-8 levels were also found high (9, 10) In other respiratory viral infections such as influenza, IL-1β levels were also elevated in patients with lung injury, whereas the use of antagonists of IL-1β reduced it, which indicates an important role of IL-1β in the pathogenesis of complications in viral infections such as SARS-CoV infections (11, 12).

The SARS-CoV genome encodes three ion channels proteins: E, open reading frame 3a (ORF3a), and ORF8a. E and ORF3a proteins are required for the replication of the virus (13). SARS-CoV-2 enters the alveolar epithelial cells leading to their injury and apoptosis. Then the apoptotic pneumocytes release danger- and pathogen-associated molecular patterns which trigger inflammasomes of alveolar macrophages. NLRP3 inflammasome is also triggered by SARS-CoV-2 through other pathways: a) through the proteins E, 3a, and 8b of SARS-CoV, b) after the binding of the spike protein of the virus to ACE2 receptors of pneumonocytes, c) after the activation of the renin–angiotensin–aldosterone system (RAAS) leading to elevated levels of angiotensin II, which following the binding to angiotensin I receptor activates inflammasome, d) through the activation of the complement cascade by the N proteins of the SARS-CoV-2, which results in the release of complement fragments (C3a and C5a anaphylatoxins) which may upregulate inflammasome in cells (14). NLRP3 inflammasome activation leads to a release of IL-1β, IL-18, and TNF-α, initiating a cascade of secretion of proinflammatory cytokines, cell apoptosis, and tissue injury (15). IL-1β leads to overproduction of INF-1*γ* by NK cells leading to hemophagocytosis. Cytokine storm may cause dysfunction of NK and CD cells and may include other proinflammatory cytokines or chemokines such as IL-17, IL-21, IL-22, IL-6, TNF-α, chemokine ligand 10 and 2, IL-2R, IL-8, IL-10, which may also participate in ARDS/ALI and multi-organ failure (4). NLRP3 activation promotes pyroptosis, a programmed cell death of immune cells which increases intracellular pathogen clearance. After pyroptosis, IL-1β and IL-18 are secreted again stimulating inflammasome. Thus, pyroptosis has a double role for innate immunity. It protects from infections, and on the other hand, it may lead to chronic inflammation, too (16).

In sHLH/MAS, immunomodulation may be beneficial. Tocilizumab (IL-6 receptor blockage, licensed for cytokine release syndrome) has already been approved for COVID-19 pneumonia in China (17), while Anakinra (IL-1β blockage) has shown surveillance benefit in patients with hyperinflammation (18).

### Pre-Existing Overactivation of NLRP3 Inflammasome in Diabetes and Its Role in COVID-19 Complications

Diabetes is shown to be among the most frequently reported comorbidities in patients with COVID-19 (1, 2, 19, 20). However, the prevalence of diabetes among these patients is shown to be 8–10%, as in the general population, which would suggest that diabetes is not a risk factor for contracting COVID-19 (21, 22), but it is associated with increased mortality and severity of disease in COVID-19 pneumonia (23). This implies that diabetic patients are at higher risk of a cytokine storm, metabolic and immune derangement.

Chronic hyperglycemia deranges immune function and increases the risk for bad outcomes due to various infections such as pneumococcal pneumonia, SARS, MERS, and H1N1 (24). Obesity and type 2 diabetes have also been considered an independent risk factor for sepsis and high mortality (25) and can lead to severe pneumonia, hepatic injury, hypercoagulation and hyperinflammation during COVID-19 infection (26, 27). A possible mechanism may be that in T2DM NLRP3 inflammasome action is upregulated leading diabetes to be a state of low-grade inflammation. Hyperglycemia is also strongly associated with an increased mortality in COVID-19 pneumonia even in patients without diabetes (28).

Glyco-lipotoxicity and oxidative stress increase products of the intermediate metabolism such as urate, cholesterol crystals, extracellular ATP, certain fatty acids (*e.g.* ceramides) and islet amyloid peptides, resulting to hyperactivation of inflammasome and caspase-1 which in turn, increases the release of IL-1β and IL-18 (29). NLRP3 inflammasome hyperactivation in diabetes, pyroptosis, and low-grade inflammation lead to a delay of INF-*γ* response and lower CD4+ and CD8+ cell numbers (30, 31). CD4+ and CD8+ decrease is associated with poor prognosis, whereas recovery of lymphocyte count coincides with clinical improvement (32, 33). Indeed, according to mouse model experiments, in the absence of CD4+ there was much more severe interstitial pneumonitis, whereas the depletion of both CD4+ and CD8+ led to an increase of neutrophils and macrophages in the lesions (34). The delay of INF-*γ* response and inflammatory monocytes and macrophage accumulation are the main causes of lethal pneumonia as it has been proved in a mouse model of SARS-CoV infection (35).

In addition, this state of low-grade inflammation in COVID-19 diabetic patients may lead to an increase of the percentage of proinflammatory memory B cells and a decrease of anti-inflammatory B-cells, resulting in an early maturation of the antibody response. In that way, Secchi M et al. have indicated that SARS-CoV-2 antigens, such as the SARS-CoV-2 spike Receptor Binding Domain, lead to a rapid development of humoral response and superimposable antibody response compared to non-diabetic patients independently of glucose levels (28, 36).

Chronic NLRP3 inflammasome overactivity leads also to lung injury and fibrosis, cardiomyopathy, and other damage (37, 38) which may make diabetic patients vulnerable to bad outcomes after an infection. Weinand B. et al. found that alveolar epithelial and endothelial capillary basal laminae were significantly thicker in samples from diabetic patients. This insult to the integrity of alveolo-capillary membrane of the lung affects the alveolar gas exchange and pulmonary function in diabetic patients (39).

Endothelial to mesenchymal transition (EndMT) may contribute to interstitial organ fibrosis, including pulmonary fibrosis in COVID-19 (40). Endothelial cells undergoing EndMT change their morphology and increase their mesenchymal proteins. This results in the breakdown of the underlying basement membrane and cell migration (41). Mechanical ventilation resulting in NLRP3 inflammasome activation facilitates EndMT. Indeed, *in vitro* studies have shown that pulmonary fibrosis and EndMT were ameliorated in NLRP3-deficient mice (42).

Furthermore, downstream activation of toll-like receptor signal regulators such as IRAK4 (interleukin-1 receptor associated kinase 4) leading to TRAF6 (tumor necrosis factor receptor-associated factor 6)-nuclear factor-κB activation can alter monocyte migration and accelerate myocarditis, too (43).

Since it is suggested that the main role of NLRP3 inflammasome in the pathogenesis of SARS-CoV-2 complications is its overactivation contributing to cytokine storm and pyroptosis (14); overactivation of inflammasome in diabetes too may also suggest a reason for the susceptibility of diabetic patients to complications and lung injury with COVID-19 infection. A common therapeutic approach to SARS-CoV-2 complications (5) and type 2 diabetes with IL-1 blockade agents (44) can strengthen the suggestion of the association of type 2 diabetes and COVID-19 complications.

### Increased Vascular Permeability and Its Role in COVID-19 Complications

Increased vascular permeability in diabetes may also contribute to the susceptibility of COVID-19 diabetic patients in ARDS and sepsis. Micro-angiopathic changes may be present in the respiratory tract of diabetic patients, interfering with gas exchanges and lung compliance (45). Endothelial glycocalyx is a major determinant of vascular permeability during inflammatory stress. Diabetic patients have impaired glycocalyx thickness due to oxidative stress which is partially restored after optimal glycemic control (46). Increased vascular permeability may promote extravascular or interstitial exudates in COVID-19 infection as has been shown in sepsis (47). In a septic state, inflammatory macrophages may release cytokines, such as IL-1β and IL-6 that induce the expression of adhesion molecules, inflammatory cell infiltration, and vascular inflammation. Endothelial cells, also, release proinflammatory cytokines which contribute to the creation and proliferation of microcirculatory lesions (48). So, the dysfunctional endothelium becomes proadhesive and procoagulant (48).

Interleukin-6, a hallmark cytokine in COVID-19 infection, promotes endothelial dysfunction (49) and also local enhancement of thrombosis (50). Recently, increased IL-6 production leading to lymphopenia and its reversal by tocilizumab, an IL-6 inhibitor, has been described in critically ill COVID-19 patients in need of mechanical ventilation (51). Indeed, tocilizumab improves endothelial function leading to an increase of effective myocardial work through a profound reduction of inflammatory burden and oxidative stress, which may explain its positive effects on COVID-19 and its complications (52). Both IL-1β and IL-6 exert detrimental effects on vascular, coronary, and myocardial function during uncontrolled inflammation such as exacerbation of rheumatoid arthritis and their inhibition by biological agents to reverse these adverse effects (53, 54).

These mechanisms may lead to susceptibility of endothelial glycocalyx during COVID-19 infection resulting in increased vascular permeability and rapid deterioration to lung alveolar exudation and pneumonitis needing mechanical ventilation (51). Glycemic oscillations in diabetes have also been suggested to increase endothelial cytokine and adhesion molecule production which, in turn, may lead to an uncontrolled extravasation of leukocytes in the alveolus during influenza infection, promoting lung damage and impairment in respiratory function (55, 56). Increased vascular lesions and endothelial inflammation put individuals with diabetes at greater risk for endothelitis in several organs whereas change of vascular change and vasoconstriction can lead to organ ischemia, tissue edema, and a procoagulant state (57). Thus, endothelial glycocalyx impairment by IL-1β, IL-6, and oxidative stress in diabetic patients may be a mechanism explaining their susceptibility to worse COVID-19 infection prognosis.

## Discussion

Diabetes is characterized by abnormally elevated glucose levels and oxidative stress. Poorly uncontrolled diabetes increases the risk of infections, hospitalizations, and mortality (58, 59). Numerous clinical studies during the 2019 influenza pandemic showed an increased susceptibility of individuals with diabetes for bad outcomes (60–62). Many recent studies have also suggested that in the new pandemic COVID-19 diabetes is one of the leading comorbidities associated with infection severity (1, 2, 19, 20). The mechanisms through which diabetes is associated with COVID-19 severity are proposed to be: the increased ACE2 receptor expressed in diabetic tissues, the dysregulation of diabetic immune system, the alveolar dysfunction, the endothelial dysfunction, and coagulopathy because of low grade inflammation and oxidative stress (31).

SARS-CoV-2 invasion activates inflammasome of the alveolar macrophages leading to further activation of the immune response which may result in a cascade of proinflammatory cytokine secretion, ARDS, and sepsis (5, 30, 31).

**Figure 1:**
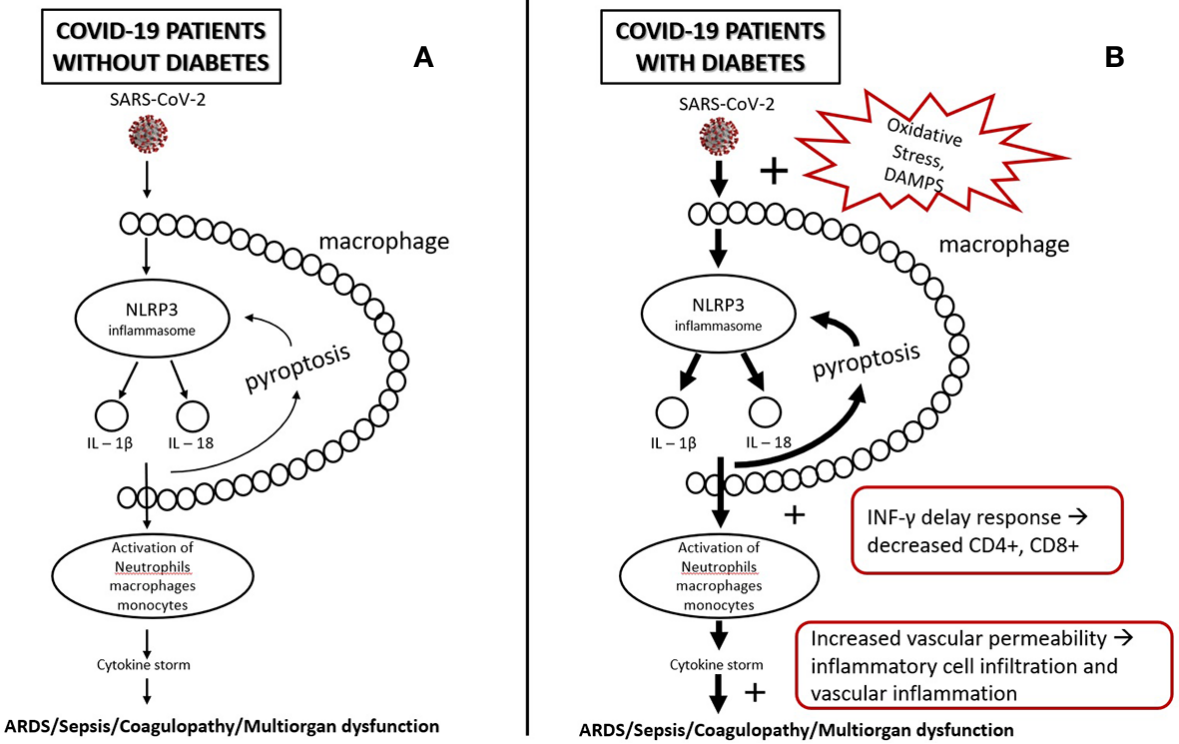
**(A)** SARS-CoV-2, after infection, triggers NLRP3 inflammasomes of alveolar macrophages. NLRP3 activation leads to a release of IL-1β, IL-18, and pyroptosis which, in turn, triggers inflammasome and leads to activation of other immune cells (e.g., neutrophils, lymphocytes, monocytes). This may initiate a cascade of secretion of proinflammatory cytokines (cytokine storm), leading to tissue injury and multiorgan failure. **(B)** In diabetes, oxidative stress and DAMPS (Damage-Associated Mollecular Patterns) overactivate inflammasomes leading to a state of low-grade inflammation and a more intense secretion of proinflammatory cytokines. Low-grade inflammation leads to a delay of INF-γ response, lymphopenia, and greater accumulation of inflammatory macrophages and monocytes, which, in combination with the increased vascular permeability and damaged glycocalyx, may increase the risk for tissue injury and multiorgan failure.

In diabetes glyco-lipotoxicity and oxidative stress increase the products of intermediate metabolism and danger-associated molecular patterns (DAMPS) which hyperactivate NLRP3 inflammasome of the macrophages which, in turn hyperactivate further the innate and acquired immunity, leading to a dysregulated immune response, a delay in INF-*γ* response, a prolonged hyperinflammatory state, and lower CD4+ and CD8+ numbers (31, 63). Furthermore, damaged glycocalyx and increased vascular permeability may promote extravascular or interstitial exudates in sepsis in COVID-19 (47, 48). Increased vascular lesions in diabetic individuals lead to greater risk of endothelitis in several organs, organ ischemia, tissue edema, and multi-organ dysfunction (32, 57) (**Figure 1**).

So, we hypothesize that the pre-existing hyperactivation of NLRP3 inflammasome, hypercytokinemia, chronic inflammation and increased vascular permeability in uncontrolled diabetes could be major contributing factors for the development of severe COVID-19 complications. Such patients could be good candidates for therapeutic intervention with colchicine, anti-IL1a, anti-IL1β, or anti-IL6 biological agents early in the course of COVID-19 infection to prevent cytokine storm, lung, and cardiovascular complications. Of course this remains to be investigated through randomized controlled studies.

## Author Contributions

All authors contributed to the article and approved the submitted version.

## Conflict of Interest

The authors declare that the research was conducted in the absence of any commercial or financial relationships that could be construed as a potential conflict of interest.

## Abbreviations

ACE-2, angiotensin-converting enzyme-2; ALI, acute lung injury; ARDS, acute respiratory distress syndrome; IL, interleukin; INF, interferon; MAS, macrophage activation syndrome; NK, natural killer; NLRP3, nucleotide-binding domain, leucine-rich-containing family, pyrin domain-containing-3; sHLH, secondary hemophagocytic lymphohistiocytosis; TNF, tumor necrosis factor.
